# Upregulation of Versican Associated with Tumor Progression, Metastasis, and Poor Prognosis in Bladder Carcinoma

**DOI:** 10.1155/2021/6949864

**Published:** 2021-02-02

**Authors:** Qi Zhang, Junxiu Wu, Xinpeng Chen, Ming Zhao, Dahong Zhang, Feng Gao

**Affiliations:** ^1^Department of Urology, Zhejiang Provincial People's Hospital, Hangzhou Medical College, Hangzhou 310014, China; ^2^Graduate Department, Zhejiang Chinese Medical University, Hangzhou 310053, China; ^3^Graduate Department, Bengbu Medical College, Bengbu, 233030, China; ^4^Department of Pathology, Zhejiang Provincial People's Hospital, Hangzhou Medical College, Hangzhou 310014, China; ^5^Department of Acupuncture & Massage, Zhejiang Provincial People's Hospital, Hangzhou Medical College, Hangzhou 310014, China

## Abstract

**Objective:**

This work analyzes the role of versican (VCAN) on bladder cancer (BLCA). Versican (VCAN) is a chondroitin sulfate proteoglycan which is important for tumorigenesis and the development of cancer. However, the expression of VCAN on human bladder cancer (BLCA) has been rarely reported.

**Methods:**

The clinical significance of VCAN in BLCA has been determined by our bioinformatics tools. Then, we performed immunohistochemical staining (IHC) and analyzed the correlation between VCAN expression and clinicopathological features.

**Results:**

The bioinformatics results reveal that a high VCAN mRNA level was significantly associated with stage, histological subtype, molecular subtype, and metastasis in BLCA. Furthermore, IHC reveals that expression of VCAN was significantly correlated with the number of tumors, invasion depth, lymph node metastasis, distant metastasis, and histological grade. Kaplan-Meier survival analysis reveals that patients with a high expression of VCAN have poor prognosis than those patients with a low expression of VCAN. According to our result from the bioinformatics database, the mechanism of VCAN in BLCA revealed that VCAN was related to FBN1 and genes of the ECM remodeling pathway (MMP1, MMP2).

**Conclusion:**

VCAN expression might be included in the process of carcinogenesis and prognosis. Hence, VCAN could be a reliable biomarker of the clinical prognosis on BLCA.

## 1. Introduction

Bladder cancer (BLCA) is one of the most prevalent malignancies of the urinary system [1]. Especially in China, the incidence and mortality rates of BLCA have gradually increased in recent years [2]. Although the diagnosis process has been improved by the development of cystoscopy, specific biomarkers for early diagnostic and prognostic assessment of BLCA are still deficient [3]. Moreover, the curative methods and five-year survival rate are limited and low for BLCA [4]. Hence, it is extremely important to explore the indicators of BLCA in order to improve the clinical treatment effect on BLCA patients.

Versican (VCAN) is a chondroitin sulfate proteoglycan located at the extracellular matrix. Previous reports have already proven that VCAN is important for the development of various diseases [5]. Through the process of direct or indirect interactions, we could see that VCAN plays significant roles in modulating cell proliferation, differentiation, adhesion, and migration [6]. In addition, VCAN is associated with the formation of a pericellular sheath which could modulate cell attachment and motility [7]. Other studies also revealed that VCAN could promote the local expansion, invasion, and formation of cells. Moreover, it could promote distant metastasis by decreasing cell-matrix adhesion as well [8, 9]. Hence, VCAN might exert function in the invasion and metastasis of tumor cells.

Recently, several studies have demonstrated that abnormal expression of VCAN was found in various cancer types such as prostate [10], breast [11], gastric [12], colorectal [13], ovarian [14], pancreatic [15], laryngeal [16], and testicular tumors [17]. Some studies reveal that VCAN plays an important role in various cancers [18, 19]. Of possibly greater concern was that VCAN has been proven to promote cell proliferation, inhibit apoptosis, and promote metastasis in the tumor [20, 21]. Stylianou et al. reveal a 140-fold increase of VCAN expression in laryngeal cancer tissue compared to normal controls [22]. VCAN was also reported to express significantly higher in severe ovarian cancer than in normal ovarian tissues [23]. Shen et al. [24] explored that the VCAN expression level was higher in gastric cancer than in adjacent normal tissues and realized that a higher VCAN level correlated with greater tumor invasion, depth, and poor prognosis. This notion was also supported by observing that increased levels of peritumoral VCAN predicted poor prognosis in patients with early-stage prostatic cancer. [25] Moreover, breast cancer and relapse with stage I node-negative were associated with the level of VCAN accumulated in peritumoral stroma as well [26]. These above studies reveal that VCAN might have an oncogenic role in tumors.

In contrast to the oncogenic role of VCAN, de Wit et al. reveal that protein expression of VCAN predicted better clinical outcome for colon cancer patients with stages II and III [27]. Voutilaine et al. also reported that a higher expression of VCAN in epithelial cells was correlated with a longer survival time, while a higher level of VCAN in the tumor stroma was an indicator for poor prognosis [14]. It showed that VCAN seems to exert its function by interacting with different types of proteins in a tissue-specific manner.

However, the physiological role of VCAN in BLCA still needs to be explored. The purpose of our study was to investigate VCAN expression in BLCA, to determine the relationship with clinicopathological factors, and also to focus on its prognostic significance. At first, we analyze the VCAN expression in human BLCA by combining the bioinformatics analysis of publically available databases. Then, we confirmed the role of VCAN in 417 cases of BLCA in a Chinese population with immunohistochemical staining. According to our presented study and this broad analysis, VCAN may represent valuable candidate biomarkers on BLCA prognosis and treatment.

## 2. Materials and Methods

### 2.1. Identification of VCAN Expression in BLCA Based on Bioinformatics Database

UALCAN (http://ualcan.path.uab.edu) was used for analyzing the mRNA level of VCAN in BLCA. GEPIA (http://gepia.cancer-pku.cn/) was used to perform customizable functionalities, and the data are from TCGA and GTEx data. The prognostic value of the VCAN mRNA level was also analyzed by the above database.

Then, the prognostic value of the VCAN mRNA level in 402 cases of BLCA was assessed by using the OncoLnc database (http://www.oncolnc.org), and the survival analysis was performed using cutoff values of the median of the VCAN expression in BLCA patients.

Furthermore, STRING database was used to explore the interaction between VCAN and other related proteins, the Comparative Toxicogenomics Database (CTD) was used to explore the gene-drug interaction as well, and drugs or chemicals which could affect VCAN expression were searched in the CTD database.

### 2.2. Patient Samples and Construction of Tissue Microarray (TMA)

417 cases of paraffinized specimens were collected from BLCA patients who underwent curative resection from 1998 to 2010 at the Department of Surgery (Zhejiang Provincial People's Hospital, Hangzhou, China). Those samples were used for construction of a tissue microarray (TMA).

The patient samples consisted of 366 males and 51 females aged 35–79 years (median age 63.3 years), and there were 147 cases with a low histological grade and 270 cases with a high histological grade according to the World Health Organization pathological classification of tumors. Meanwhile, 35 cases of patients presented with distant metastasis and 382 cases without distant metastasis.

The end point was overall survival (OS), which was calculated from the date of operation performed to the cutoff point during follow-up period (December 2015) or the date of death. All patients did not receive chemotherapy or radiotherapy before the operation.

The construction of TMA was performed by the Shanghai Outdo Biotech Company (Shanghai, China), and the process of the construction of TMA was described as follows: Firstly, the tissue slides were stained with hematoxylin and eosin to select the most representative tissue block from each case. Then, the targeted areas of each donor tissue block (0.6 mm in diameter) were punched and arranged in empty recipient paraffin blocks of 35 × 18 mm using a tissue microarray instrument (MTA-1, Beecher Instruments, Silver Spring, MD, USA). As each core is placed into the recipient block, the block identification number should be noted on the array map. Then, the TMA blocks were fused in 50°C-52°C for 6 hours and immersed into the melted wax quickly to coat the surface with wax. After that, the TMA blocks were stored in 4°C after natural cooling. After the TMAs were constructed, 4 *μ*m sections were cut from the TMA blocks to generate TMA slides. The slides were dried overnight at room temperature and then baked at 60°C for 20 min before being stored.

The project was approved by the ethics committee of the Zhejiang Provincial People's Hospital, and written informed consent was obtained from all participants involved.

### 2.3. Immunohistochemistry

At first, sections were deparaffinized by dimethylbenzene and rehydrated in descending series of ethanol. Then, the slides were incubated with 3% H_2_O_2_ at room temperature to eliminate endogenous peroxidase. Antigens were retrieved by maintaining the temperature between 92°C and 98°C in a 0.01 M citrate buffer (pH 6.0) for 4 mins. Subsequently, slides were then incubated with 10% (vol/vol) normal goat serum for 20-30 mins at room temperature in order to reduce nonspecific reactions.

Then, the slides were added with primary rabbit anti-VCAN polyclonal antibody (ab19345, Abcam, Massachusetts, US) with a 1 : 300 dilution in phosphate-buffered saline at 4°C overnight. The next day, the slides were incubated with biotin-labeled secondary antibody followed by horseradish peroxidase-linked antibody (Zymed, San Francisco, CA) for 30 mins at room temperature after rinsing with phosphate-buffered saline (PBS). Then, the sections were stained with 3,3-diaminobenzidine, and the nuclear was counterstained by hematoxylin. After that, the slides were rehydrated in ascending series of ethanol and then covered by neutral resin.

### 2.4. Evaluation of Immunostaining Intensity

The assessment of the immunostaining was conducted in a semiquantitative way, using an immunoreactivity score by two expert pathologists under light microscope without knowing the clinical data. The experts scored independently according to the intensity of cell staining and the proportion of tumor cells stained.

We divided the results into grades with no expression (intensity 0), low intensity of expression (intensity 1), medium intensity of expression (intensity 2), and high intensity of expression in the tumor cell (intensity 3). Staining intensity was scored according to the following criteria: 0 (no staining), 1 (weak staining, light yellow), 2 (moderate staining, yellow brown), and 3 (strong staining, brown). Positive stained areas of VCAN-positive cells were expressed as the percentage of whole cancer areas and were scored as follows: 0 for no cytoplasm expression, 1 for ≤25% positive cancer cells, 2 for 26-50% positive cancer cells, 3 for 51-75% positive cancer cells, and 4 for ≥76% positive cancer cells.

According to the above data, the composite score of the staining was obtained by the product of the intensity and the proportion scores. Thus, the total values were ranged from 0 to 12. In the present study, we defined the score ≤ 5 as the low expression of VCAN and the score > 6 as the high expression of VCAN for the next evaluation.

### 2.5. Statistical Analysis

The Statistical Package for the Social Sciences (version 13.0; SPSS Inc., Chicago, IL) was used to perform all statistical analyses. *χ*^2^ or Fisher's exact test was performed to analyze the categorical data. The Kaplan-Meier method accompanying the log rank test was performed to estimate survival analysis. Meanwhile, multivariate survival analysis was performed to assess predictors related to prognosis using the Cox proportional hazard regression model. For all tests, *P* values were obtained from two-tailed statistical tests, and *P* values less than 0.05 were considered statistically significant.

## 3. Results

### 3.1. The Upregulation of VCAN mRNA Level Was Correlated with Progression of BLCA

**Figure 1:**
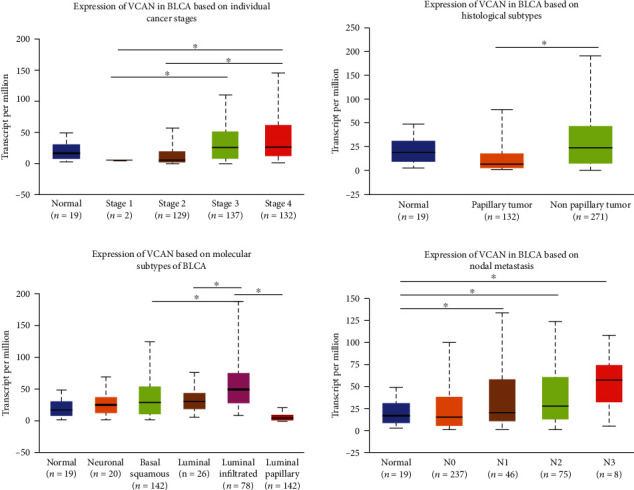
The *VCAN* mRNA level was associated with stage, histological subtype, molecular subtype, and node metastasis of BLCA used by the UALCAN database. N0: no regional lymph node metastasis; N1: metastases in 1 to 3 axillary lymph nodes; N2: metastases in 4 to 9 axillary lymph nodes; N3: metastases in 10 or more axillary lymph nodes. ^∗^*P* < 0.05.

In order to investigate the role of VCAN in human BLCA progression, we first performed bioinformatics analysis with published datasets to detect the VCAN mRNA levels in BLCA by the UALCAN database, and the results reveal that the VCAN mRNA level was significantly higher in patients with BLCA, and a high mRNA level of VCAN was associated with stages, histological subtypes, molecular subtypes, and node metastasis (Figure 1). The patients with a more advanced stage result in a higher mRNA level of VCAN, and the mRNA level of VCAN was significantly higher in patients with a nonpapillary tumor than in patients with a papillary tumor. Moreover, the mRNA level of VCAN was significantly higher in BLCA patients with luminal infiltration than other molecular subtypes, and node metastasis is also associated with the mRNA expression level of VCAN in BLCA.

### 3.2. Relationship between VCAN Expression and Clinicopathological Parameters of BLCA

**Figure 2:**
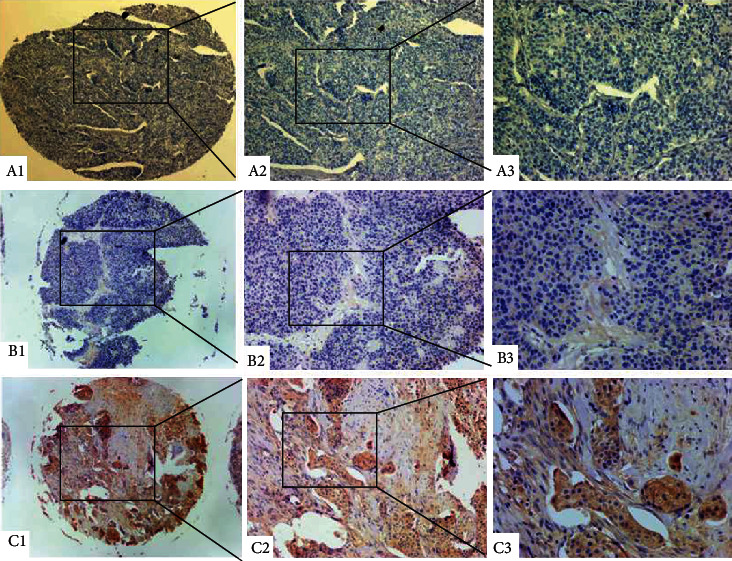
Immunohistochemical staining for VCAN in cancerous bladder tissue. (A1–A3) Negative staining in urinary bladder carcinoma. (B1–B3) Weak staining in non-muscle-invasive urinary bladder carcinoma. (C1–C3) Strong staining in muscle-invasive urinary bladder carcinoma. Magnification: the original magnification ×40 (A1–C1), ×100 (A2–C2), and ×200 (A3–C3).

Immunohistochemical staining results reveal that VCAN was most localized in the cytoplasm (Figure 2). Then, the relationship between VCAN protein levels and clinicopathological parameters of BLCA was investigated (Table 1). The results showed that the rate of high expression of VCAN in BLCA was 70.3% (293/417), and high expression of VCAN was correlated with the number of tumors, invasion depth, lymph node metastasis, distant metastasis, and histological grade. But no association was presented between VCAN expression and other parameters such as age, gender, size, and lymph invasion.

High expression of VCAN was detected in 48 of 88 (54.5%) cases of patients with a single tumor and in 245 of 329 (62.5%) cases of patients with multiple tumors, which showed a significant difference (*χ*^2^ = 13.19, *P* < 0.001, Table 1). The percentage of the VCAN high expression was 74.2% (264/356) in patients with invasion depth T2-T4, which was higher than that in patients with invasion depth T0-T1 (47.5%, 29/61) (*χ*^2^ = 17.68, *P* < 0.001). High expression of VCAN was detected in 66.0% (208/315) BLCA patients without lymph node metastasis, which was lower than that with lymph node metastasis (83.3%, 85/102, *χ*^2^ = 11.039, *P* = 0.001). High expression of VCAN was detected in 262 of 382 (68.6%) cases of patients without distant metastasis, and in 31 of 35 (88.6%) cases of patients with distant metastasis, which showed a significant difference (*χ*^2^ = 6.13, *P* = 0.013). The percentage of the VCAN high expression was 64.6% (95/147) in patients with a low histological grade, which was lower than in patients with a high histological grade (73.3%, 198/270) (*χ*^2^ = 10.58, *P* = 0.005).

### 3.3. Prognostic Significance of VCAN Expression in BLCA

**Figure 3:**
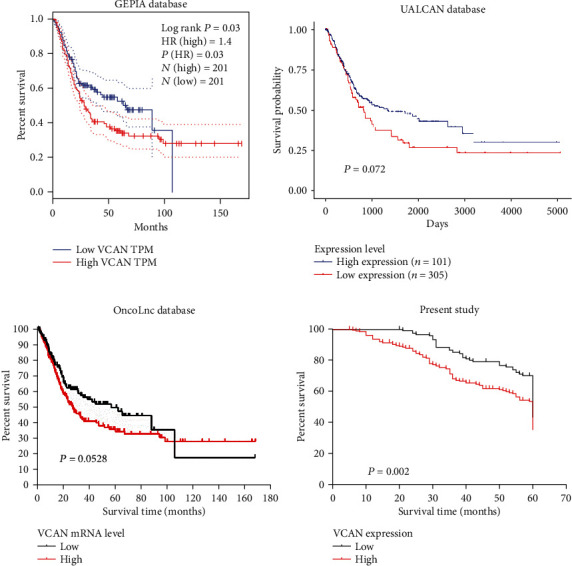
Kaplan-Meier survival curves of bladder carcinoma patients with different levels of VCAN expression.

To define the clinical prognostic value of the VCAN, we explored the prognostic significance of VCAN by the GEPIA database, OncoLnc database, and UALCAN database. The Kaplan-Meier curve performed with the GEPIA and UALCAN databases showed that the survival rate of patients with a high mRNA level of VCAN was lower than those with a low mRNA level of VCAN (*P* = 0.03 and *P* = 0.072, respectively). The Kaplan-Meier curve performed with the OncoLnc database revealed that the difference was not significant (log rank, *P* = 0.0528, Figure 3).

In our presented study, the Kaplan-Meier survival analysis reveals that the mean survival time in patients of BLCA with a VCAN low expression was 53.79 ± 1.03 months and 46.99 ± 1.09 months for those with a high expression of VCAN. Meanwhile, the 5-year cumulative survival rates in BLCA patients with a low VCAN expression were 70.3% and 53.5% in those with a high expression of VCAN. Obviously, BLCA patients with a high expression of VCAN reveal poor prognosis than those with a low expression of VCAN (Figure 3, log rank test, *χ*^2^ = 9.690, *P* = 0.002).

Univariate analysis indicated that the factors significantly associated with survival were tumor size (*P* = 0.037), lymph node metastasis (*P* = 0.002), lymphovascular invasion (*P* = 0.048), distant metastasis (*P* = 0.002), depth of invasion (*P* < 0.001), TNM stage (*P* < 0.001), and VCAN expression (*P* = 0.003) (Table 2). The clinicopathological parameters that were correlated with survival in the univariate analysis were included in the multivariate analysis. Hence, tumor size, lymph node metastasis, lymphovascular invasion, distant metastasis, depth of invasion, TNM stage, and VCAN expression were included in Cox's proportional hazard regression model. It was indicated that distant metastasis (*P* = 0.042), depth of invasion (*P* = 0.036), TNM stage (*P* < 0.001), and VCAN expression (*P* = 0.001) were independent prognostic factors for patients with BLCA, whereas tumor size, lymph node metastasis, and lymphovascular invasion were not (Table 2).

### 3.4. Mechanism of VCAN in BLCA Based on Bioinformatics Database

**Figure 4:**
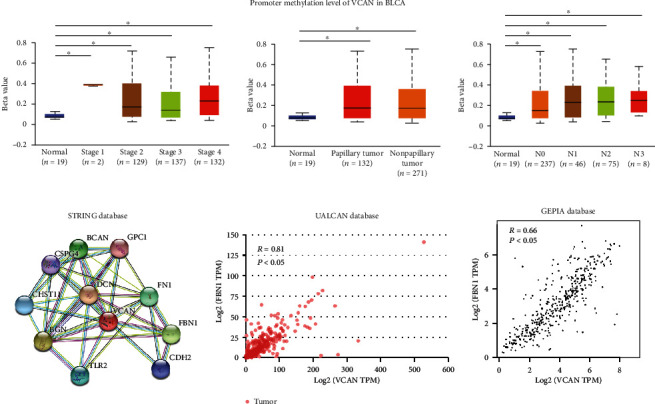
(a) Hypomethylation of VCAN was associated with progression of BLCA. (b) VCAN is involved in the interaction network in cell adhesion for the ECM remodeling pathway, and the mRNA expression level of VCAN in BLCA was positively correlated with FBN1.

Furthermore, we also explored the potential mechanism of VCAN being involved in cancer progression, and we realized that hypomethylation of VCAN was associated with stage, histological subtypes, and metastasis of BLCA (Figure 4(a)). Next, we constructed the protein-protein interaction (PPI) network from the STRING database between VCAN and other related proteins, and we found that fibrillin 1 (FBN1) was correlated with VCAN. FBN1 is the primary component of microfibrils at the extracellular matrix, which might be involved in cancer progression. We detected the correlation between VCAN and FBN1 with the UALCAN and GEPIA databases, and the results showed that VCAN correlated with FBN1 (*R* = 0.81 and *R* = 0.66, respectively, Figure 4(b)).

**Figure 5:**
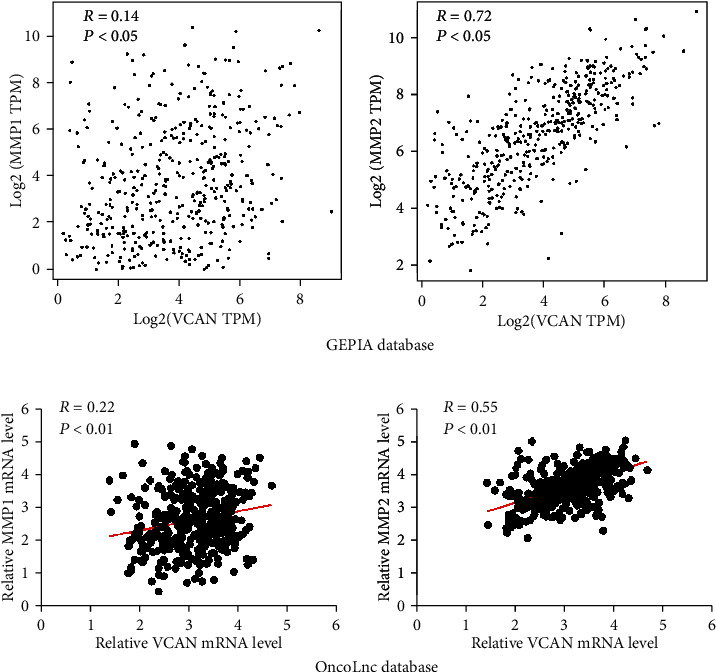
(a) The mRNA expression level of *VCAN* in BLCA was positively correlated with MMP1 and MMP2 performed by the GEPIA database. (b) The mRNA expression level of *VCAN* in BLCA was positively correlated with MMP1 and MMP2 performed by the OncoLnc database.

The PPI network also reveals that proteins were related with cell adhesion of the ECM remodeling pathway (DCN, FN1 etc., Figure 4(a)), suggesting that VCAN may be involved in tumor progression which is an important ECM component through the ECM remodeling pathway. Next, we also extracted the mRNA level of MMP1 and MMP2 in the OncoLnc and GEPIA databases and analyzed the correlation between VCAN and MMP. The results showed VCAN was directly correlated with MMP1 and MMP2 (Figure 5).

**Figure 6:**
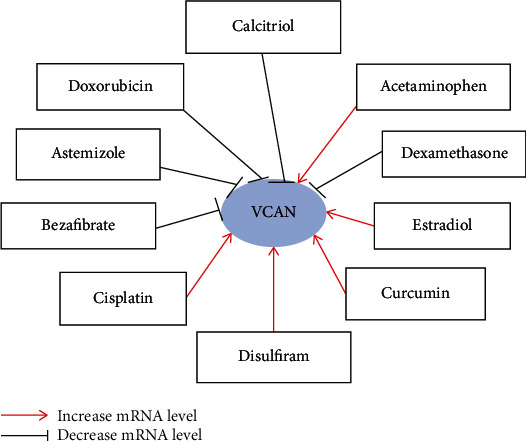
Gene-drug interaction based on the Comparative Toxicogenomics Database. The interaction network showed that several drugs or chemicals such as acetaminophen, cisplatin, and curcumin could increase the mRNA level of *VCAN*. However, doxorubicin and dexamethasone result in decreased expression of *VCAN* mRNA.

In the next step, in order to explore how available chemicals or drugs could influence VCAN expression, we constructed a gene-drug interaction network based on the Comparative Toxicogenomics Database (CTD) (Figure 6). This network revealed that several drugs or chemicals could influence the expression of VCAN. For example, several drugs or chemicals such as acetaminophen, cisplatin, and curcumin could increase the mRNA level of *VCAN*. However, doxorubicin and dexamethasone result in a decreased expression of *VCAN* mRNA.

## 4. Discussion

The stroma around solid tumors consists of specific extracellular matrix (ECM) components, which plays important roles in the microenvironment of primary and secondary tumor sites [28]. Meanwhile, the tumor microenvironment not only responds to tumor epithelial cells and supports carcinogenesis but actively contributes to tumor progression and metastasis [29]. In recent reports, a direct relationship between growth factor-mediated signaling and modulating extracellular matrix (ECM) components was identified [30]. VCAN was a member of the large aggregating chondroitin sulfate proteoglycan (CSPG) family; it is an important ECM component, which has been implicated in tumor progression [6].

As far as it is concerned, our study reported clinical data for the prospective power of VCAN expression in BLCA, and we found that the VCAN mRNA level was significantly higher in patients with infiltrating BLCA than in those with superficial BLCA based on the bioinformatics analysis. The Kaplan-Meier curve showed that the survival rate of patients with a high mRNA level of VCAN was lower than those with a low mRNA level of VCAN, although the difference between them was not significant. But we speculate that the dysregulated VCAN expression may contribute to bladder development and/or progression.

Although malignant cells can synthesize VCAN, the mechanism and functional role of epithelial VCAN expression remain to be elucidated [31]. Hence, more studies are required to clarify this issue. Then, we confirmed the role of VCAN in 417 cases of BLCA from a Chinese population with immunohistochemical staining. The results revealed that a high expression of VCAN correlated with the number of tumors, invasion depth, lymph node metastasis, distant metastasis, and histological grade. The overexpression of VCAN in cancer has also been reported to be associated with tumor progression [24]. These results suggest that VCAN is an important molecule in the progression of these malignant tumors.

Touab et al. [31] reported that cell-associated VCAN is involved in the progression of melanomas. However, epithelial VCAN expression is reported to be significantly higher in early-stage epithelial ovarian cancer [14], and tumor cell-associated VCAN is not significantly associated with clinicopathological factors in NSCLC [32]. It reveals that the mechanism and functional role of VCAN in tumors still remain unclear, and VCAN seems to exert its function in a tissue-specific manner.

Previous studies have shown that increased levels of VCAN are associated with poor prognosis of patients in a wide range of malignant tumors [33, 34]. However, a study reported the opposite effect that VCAN expression in epithelial cells was correlated to a longer survival time [14]. In the present study, the Kaplan-Meier curve analysis of 417 cases of BLCA showed that patients with a high expression of VCAN showed a poorer prognosis than those with low expression of VCAN, which suggested that VCAN upregulation may contribute to the prognosis of BLCA patients. In prostate cancer, increased concentration of stromal VCAN is an independent predictor of outcome for patients with moderately differentiated tumors [25]. In a similar way, peritumoral VCAN was a strong predictor of relapse-free survival in breast cancer [26]. An increased level in the expression of stromal VCAN has also been previously reported; it was correlated with a poor prognosis in some types of cancers [32, 35]. Thus, VCAN may be applied as a biomarker for malignancy and monitoring prognosis in BLCA clinically.

It is well known that the tumor environment is one of the major factors that determine the behavior of malignant cells. A decrease in the adhesive ability of tumor cells at the invasive foci has been noted in a number of human cancers [36, 37]. The three-dimensional structure of the extracellular matrix (ECM) was reported to regulate cell migration, differentiation, and proliferation, which then regulate biological development and tissue repair and/or alternatively cancer progression [38]. Remodeling of the extracellular matrix (ECM) could be made through the altered expression of molecules integrated in the functional network. For example, cell-to-cell and cell-to-matrix interactions are essential for local tumor cell invasion and metastasis. The study also reported that modifications of the ECM composition during tumor development may be crucial for tumor initiation and development [38]. Gorter et al. revealed that VCAN expression in the stromal compartment of cervical cancers results in reduced numbers of intraepithelial CD8-positive T cells and enhanced local invasion [39]. Our present study revealed a high expression of VCAN which correlated with invasion depth, lymph node metastasis, and distant metastasis. We suspect that VCAN may play a role in the cell-ECM adhesion interactions during cancer progression and may be used as a prognostic marker and therapeutic target for the treatment of the disease. We found that that VCAN may be involved in tumor progression as an important ECM component through the ECM remodeling pathway as well. Furthermore, we constructed a gene-drug interaction network, which suggested that several drugs or chemicals such as doxorubicin and dexamethasone could result in decreasing the mRNA level of VCAN. Several drugs or chemicals such as cisplatin and curcumin could result in increasing the mRNA level of *VCAN*, which suggested that clinical treatment of bladder cancer with these drugs may induce poorer prognosis through increasing the VCAN level. Therefore, we declared that many available chemicals are efficient in correcting the abnormal gene expression of VCAN, and this could provide more strategies for BLCA therapy.

In conclusion, the present study provides novel evidence regarding VCAN expression in BLCA and its involvement in carcinogenesis and progression. The results highlight the independent contribution of VCAN overexpression towards poorer outcomes for patients with BLCA, which revealed that the VCAN expression may contribute to the aggressive biological behavior of BLCA and could be a reliable marker for clinical prognosis.

## Figures and Tables

**Table 1:** Relationship of VCAN expression with pathological parameters of bladder cancer.

Clinical parameters	VCAN expression			
	Low	High	*χ* ^2^	*P*
Gender			0.859	0.354
Male	106 (29.0%)	260 (71.0%)		
Female	18 (35.3%)	33 (64.7%)		
Age (yrs)			0.823	0.364
<60	42 (27.1%)	113 (72.9%)		
≥60	82 (31.3%)	180 (68.7%)		
Size			3.597	0.058
<3 cm	71 (34.0%)	138 (66.0%)		
≥3 cm	53 (25.5%)	155 (74.5%)		
Number of tumors			13.189	0.000
Single	40 (45.5%)	48 (54.5%)		
Multiple	84 (37.5%)	245 (62.5%)		
Invasion depth			17.657	0.000
Ta-T1	32 (52.5%)	29 (47.5%)		
T2-T4	92 (25.8%)	264 (74.2%)		
Lymph node metastasis			11.039	0.001
No	107 (34.0%)	208 (66.0%)		
Yes	17 (16.7%)	85 (83.3%)		
Distant metastasis			6.129	0.013
No	120 (31.4%)	262 (68.6%)		
Yes	4 (11.4%)	31 (88.6%)		
Lymphovascular invasion			3.648	0.056
Negative	119 (31.0%)	265 (69.0%)		
Positive	5 (15.2%)	28 (84.8%)		
Histological grade			10.577	0.005
Low grade (superficial)	52 (35.4%)	95 (64.6%)		
High grade (infiltrating)	72 (26.7%)	198 (73.3%)		
TNM stage			4.842	0.184
I	33 (31.4%)	72 (68.6%)		
II	24 (24.0%)	76 (76.0%)		
III	39 (27.9%)	101 (72.1%)		
IV	28 (38.9%)	44 (61.1%)		

**Table 2:** Multivariate analysis of the correlation between clinicopathological parameters and survival time of patients with bladder cancer.

Parameters	Univariate analysis			Multivariate analysis		
	HR	95% CI	*P*	HR	95% CI	*P*
Age	1.281	0.941-1.742	0.115	NA		
Gender	1.385	0.920-2.086	0.119	NA		
Tumor size	1.367	1.019-1.835	0.037	1.047	0.777-1.411	0.764
Tumor number	1.358	0.942-1.959	0.101	NA		
Lymph node metastasis	1.694	1.209-2.373	0.002	0.967	0.669-1.398	0.859
Lymphovascular invasion	1.708	1.004-2.903	0.048	1.289	0.751-2.212	0.357
Distant metastasis	2.218	1.343-3.664	0.002	1.742	1.019-2.977	0.042
Depth of invasion	2.611	1.538-4.431	<0.001	1.787	1.038-3.076	0.036
Histological grade	1.352	0.982-1.860	0.064	NA		
TNM stage	1.805	1.554-2.098	<0.001	1.867	1.578-2.210	<0.001
VCAN expression	1.611	1.172-2.214	0.003	1.820	1.292-2.565	0.001

## Data Availability

The data used to support the findings of this study are included within the article.
